# Simultaneous Second Harmonic Generation of Multiple Wavelength Laser Outputs for Medical Sensing

**DOI:** 10.3390/s110606125

**Published:** 2011-06-07

**Authors:** Seung Nam Son, Jae-Jin Song, Jin U. Kang, Chang-Seok Kim

**Affiliations:** 1 Department of Cogno-Mechatronics, Pusan National University, Busan, 609-735, Korea; E-Mail: nami-new@hanmail.net; 2 Department of Otorhinolaryngology, Seoul National University Bundang Hospital, Kyungki, 463-707, Korea; E-Mail: jjsong96@hanmail.net; 3 Department of Electrical and Computer Engineering, Johns Hopkins University, Baltimore, MD 21218, USA; E-Mail: jkang@jhu.edu

**Keywords:** hemoglobin concentration, oxygen saturation, second harmonic generation, fiber Bragg gratings, Er-doped fiber laser, multiple wavelength

## Abstract

Multiple wavelength light sources in the medical spectral window region are useful for various medical sensing applications in tissue by distinguishing the absorption and scattering coefficients optically. We propose a simultaneous second harmonic generation of multiple wavelength fiber laser output using parallel channels of periodically-poled lithium niobate (PPLN) waveguides. High intensity dual wavelength lasing output is experimentally realized with two tunable fiber Bragg gratings of 1,547.20 nm and 1,554.48 nm for the efficient conversion to the half wavelengths, 773.60 nm and 777.24 nm, by using two parallel PPLN channels. Compared with a conventional dual second harmonic generation (SHG) configuration based on two different input wavelengths from each independent light source, this method has a relatively higher efficiency to align the input light beam into the adjacent parallel PPLN channels simultaneously. The use of fiber lasers offers several advantages since they are relatively inexpensive, provide high power in excess of tens of watts, are widely tunable, and can produce pulses from milliseconds to femtoseconds.

## 1. Introduction

Multiple wavelength light sources are useful for spectroscopic sensing applications including medical diagnostics, agrochemical analysis, as well as cognitive neuroscience research. For example, since the absorption of light is minimum around 700 to 900 nm for blood-perfused biological tissue, the light in this so-called ‘medical spectral window’ region penetrates deeply into tissues allowing for non-invasive investigations. The optical penetration depth of tissues is limited by the absorption of hemoglobin at shorter wavelengths, and by the water absorption at longer wavelengths. For non-invasive optical sensing of oxygenated and de-oxygenated hemoglobin concentration in tissue regions, at least two different wavelengths around 700–900 nm are required to distinguish the absorption and scattering coefficients of the medium [1,2].

Fiber sources for near-infrared (NIR) wavelength region around 1,550 nm were rapidly developed during the last decades and are capable of producing high power single or multiple wavelengths simultaneously [3]. Furthermore, they are widely tunable and can be configured to produce pulses with a wide range of pulse widths and duty cycles. However, there are no relatively inexpensive comparable tunable sources around the fiber lasers’ double frequency region, ∼775 nm. Second harmonic generation (SHG) is a nonlinear optical process that generates twice the frequency of the incident light when the high intensity beam interacts with a nonlinear optical crystal. When a shorter wavelength SHG beam can be generated from a longer wavelength near-infrared fiber laser beam, it could be useful for a wide range of medical diagnostics [4].

In this research, we propose a simultaneous SHG in medical spectral window using a multiple wavelength fiber laser and a periodically-poled lithium niobate (PPLN) containing parallel waveguides. The multiple wavelengths of the seed fiber source are carefully selected using a pair of intracavity tunable fiber Bragg grating (FBG) filters. The output intensity of the multiple wavelength source is amplified by an additional high power erbium-doped fiber amplifier (EDFA). For the high efficiency conversion of multiple wavelengths simultaneously, the PPLN was be integrated into parallel channels using a phase matched waveguide structure.

## 2. Experimental Setup and Simulation

**Figure 1.**
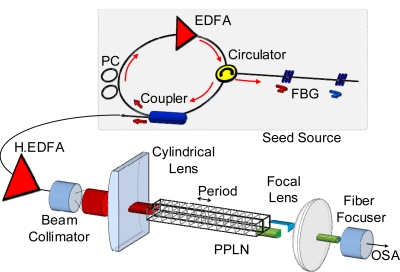
Experimental setup.

**Figure 2.**
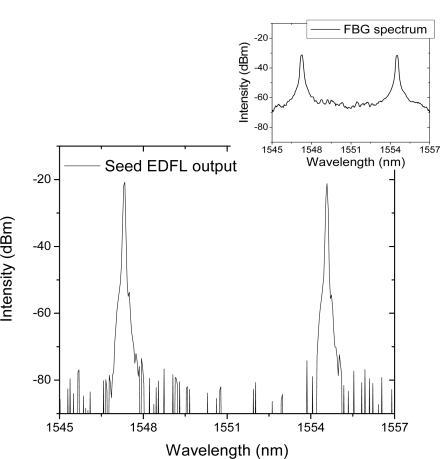
Spectrum of Seed EDFL output. Inset shows the spectrum of two tunable fiber Bragg gratings.

The experiment was performed with a dual wavelength Er-doped fiber laser, a high power EDFA, and two parallel PPLN channels in a temperature-controlled oven. Figure 1 shows the experimental setup. The seed source was configured with a dual wavelength ring laser system using two FBGs with center wavelengths of 1,547.20 nm and 1,554.48 nm, respectively. As shown in Figure 2, the lasing wavelengths of the seed source are exactly determined from the spectrum of two FBGs. These two lasing wavelengths can be controlled accurately within a few nm range by the external pulling stretcher on each FBG filter. The center peak wavelength of FBG is linearly shifted to a longer wavelength region when we apply a pulling strain on both end positions of the FBG component. For the high intensity input to the energy efficient CW PPLN conversion, a 33 dBm EDFA is additionally prepared at the output of a dual wavelength laser source.

The free space optical beam of fundamental output was optimally shaped to a line beam using a cylindrical lens of 8 cm focal length because it was necessary to simultaneously transmit through two parallel channels of the PPLN. In this experiment, a circular beam with a diameter of 1.6 mm from a beam collimator (HPUCO-23A-1300/1550-S-11AS) was shaped to the oval line beam with a height of 0.14 mm and width of 1.6 mm at the focal position of 8 cm from the cylindrical lens (LJ1105L1, Thorlabs). Since each PPLN channel has a height of 0.5 mm, width of 0.2 mm and length of 20 mm, the simultaneous SHG can be easily obtained as positioning multiple parallel channels of the PPLN waveguide at the central focal position of a cylindrical lens. The multi-period PPLN device used in this experiment (97-02355-01, Crystal Technology) includes 10 parallel PPLN channels with sequential poling periods of 18.6, 18.8, 19.0, 19.2, 19.4, 19.6, 19.8, 20.0, 20.2 and 20.4 μms. Since there is a separation space of 1.06 mm between each channel, the whole width of the PPLN waveguide device is 11.5 mm.

The quasi-phase-matching condition can be simply described with the following equation:

(1) $$\Delta k=k_{2\omega }-2k_{1\omega }$$

where $\Delta k=\frac{2\pi }{\Lambda }$, $k_{2\omega }=\frac{2\pi n_{2\omega }(T)}{\lambda_{2\omega }}$, and $k_{1\omega }=\frac{2\pi n_{\omega }(T)}{\lambda_{\omega }}$. Here, Λ is the poling period in a PPLN channel, *λ_ω_* is the input light wavelength, and *λ_2ω_* is the converted SHG light wavelength. The refractive indexes, *n_2ω_* and *n_ω_*, at both wavelengths depend on the temperature, *T*, of the PPLN crystal.

**Figure 3.**
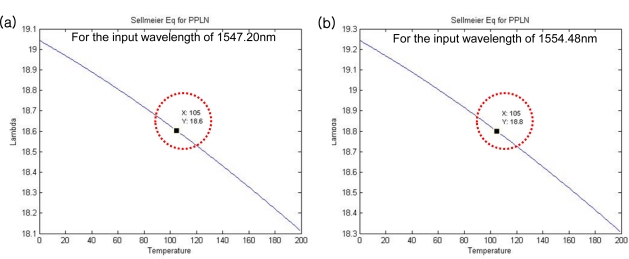
Simulation between the temperature (°C) and PPLN period (Λ) for each input wavelength of **(a)** 1,547.20 nm and **(b)** 1,554.48 nm.

From the above relation, it is clear that there exists only one pair of input light wavelengths, *λ_ω_*, and PPLN periods, Λ, under a certain temperature condition, *T*. Thus, in order to generate two SHG wavelengths simultaneously using two adjacent PPLN periods, it is necessary to find an optimal condition such that two input wavelengths in a single light beam satisfy this quasi-phase-matching condition under the same temperature simultaneously. Compared with a conventional dual SHG configuration based on two different input wavelengths from each independent light source, this method has a relatively higher efficiency to align the input light beam into the adjacent parallel PPLN channels simultaneously. We performed a simulation in MATLAB to find this optimal condition using an iterative Sellmeier equation code. As a result, the optimal poling periods of 18.6 μm and 18.8 μm were obtained for the input wavelengths of 1,547.20 nm and 1,554.48 nm, respectively, under the phase-matching temperature of 105 °C [5,6]. Figure 3 shows the simulation result of this optimization process.

For the output beam through PPLN, a dichroic filter (850FG07-25, Andover Corp.) was used to separate the 775 nm region SHG beam from the 1,550 nm region fundamental beam. The spectrum of SHG was measured using an optical spectrum analyzer (OSA) by the focus collimation from the free space beam to the optical fiber.

## 3. Experiment Results

**Figure 4.**
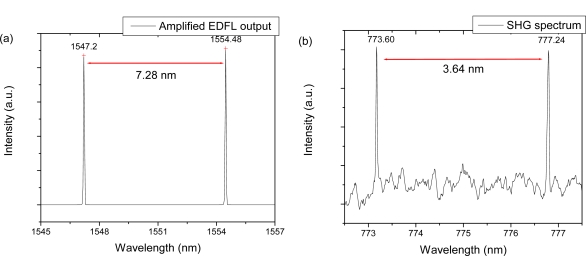
**(a)** Spectrum of amplified EDFL output as a fundamental NIR beam. **(b)** Spectrum of converted SHG beam.

The spectrum from the dual wavelength laser source is represented in Figure 2. Each peak is located at 1,547.20 nm and 1,554.48 nm, respectively, as expected from the tunable FBG specification. After the 33 dBm high power EDFA, the maximum output power was 1.5 W for the fundamental beam from the free space collimator output in the experiment. As shown in Figure 4(a), the spacing of the two peaks is still the same as that of the seed source, 7.28 nm. The intensity scale is not noted in Figure 4 because only a small part of the high power output is tapped using a directional coupler to monitor the spectral information with OSA for damage protection purposes. These two wavelength peaks of one line-shaped oval beam are converted simultaneously to the SHG wavelength through two parallel PPLN channels with a period at 18.6 μm and 18.8 μm, respectively.

Figure 4(b) shows the converted spectrum of the dual peak SHG beam from the dichroic filter high power dual wavelength laser source. Each peak is at 773.60 nm and 777.24 nm, respectively, which corresponds to half the wavelength of the input wavelength. The spacing is measured to 3.64 nm, which is also half the spacing of the dual wavelength laser beam. The full wave half maximum (FWHM) of each peak is around 0.04 nm and the power of converted output beam is measured to be less than 10 mW. Since the laser is a continuous wave (CW) mode signal, the conversion power efficiency remained at the level of 1% in this experiment. However, it is easily expected that the temporal variation to the pulse mode signal of the laser source can further improve the conversion efficiency more than 10% [7]. The further optimization of the conversion optics setup based on the parallel PPLN channels will be helpful to increase the conversion efficiency. By improving the multiple wavelength laser setup including multiple FBG filters, the generated number of multiple peaks in the SHG beam can be easily increased. It is also expected to change the wavelength position of multiple peaks because the input wavelengths around 1,550 nm are easily tunable using FBG filters.

## 4. Conclusions

A high-intensity dual-wavelength CW laser was built using two tunable FBG filters centered at 1,547.20 nm and 1,554.48 nm for efficient simultaneous conversion to its half wavelengths, 773.60 nm and 777.24 nm, by way of two parallel PPLN channels. This tunable multiple wavelength fiber laser source that could be easily configured to operate high power and pulsed in medical spectral window region should be useful for a wide range of medical sensing applications.
